# Surgical management of ulcerative colitis-associated colorectal cancer in a 20-year period, a single-centre study

**DOI:** 10.3389/pore.2026.1612362

**Published:** 2026-04-22

**Authors:** János Tajti, László Libor, Szabolcs Ábrahám, Zsolt Simonka, Anikó Maráz, Attila Paszt, Tamás Molnár, Judit Oláh, György Lázár

**Affiliations:** 1 Department of Surgery, University of Szeged, Szeged, Hungary; 2 Department of Oncotherapy, University of Szeged, Szeged, Hungary; 3 Division of Gastroenterology, Department of Medicine, University of Szeged, Szeged, Hungary

**Keywords:** colorectal cancer, ileal pouch-anal anastomosis, primary sclerosing cholangitis, survival rate, ulcerative colitis

## Abstract

**Introduction:**

The incidence of inflammatory bowel disease is on the rise. Inflammation that persists for years or decades may involve the risk of malignant transformation. Indeed, it is the cause of death in 15% of the UC patient population. Proctocolectomy followed by ileal pouch-anal anastomosis is the accepted surgical procedure.

**Aim:**

Our study objective was to retrospectively assess the occurrence and surgical treatment of UC-associated colorectal cancer cases in our institute and analyse survival data.

**Materials and methods:**

In our department, 39 patients (12 female and 27 male patients) underwent surgery for UC-associated colorectal cancer between 1 January 2005 and 1 January 2025. Their mean age was 55 ± 13.4 years. Risk factors for the disease, examination results, types of surgery, perioperative and long-term surgical results, and survival measures were assessed retrospectively. The latter were determined using the Kaplan–Meier analysis.

**Results:**

Thirty-nine patients were diagnosed with UC at a mean age of 35.7 ± 16.7 years, and an average of 19.4 ± 12.3 years passed between the diagnosis of UC and the first surgical intervention. Regular endoscopies were performed in only 66% of our patients. Preoperative staging confirmed distant metastases in 12 patients (30.7%). Patients underwent 34 elective and 5 emergency surgeries. The mean follow-up duration was 40.2 ± 51.7 months. Only 7 patients (17.9%) had a T1 lesion. Lymph node involvement was confirmed in 17 cases (44.5%), whereas 12 patients (30.7%) showed dissemination. Adjuvant chemotherapy was administered in 23 cases (58.9%), and follow-up was recommended for 13 patients (33.3%). During the study period, 17 of the 39 patients died. The mean survival after the surgical procedure was 98.6 months (8.2 years). Survival was significantly shorter in patients who had undergone emergency surgery, were active smokers, suffered from PSC, and lacked gastroenterological follow-up.

**Conclusion:**

Based on our experience, it is especially important for UC patients to receive close gastroenterological follow-up in specialised centres and undergo regular colonoscopies and for staff to evaluate biopsy samples properly and perform the appropriate surgical procedures in due time, preferably proctocolectomy and creation of IPAA with a minimally invasive method.

## Introduction

The incidence of inflammatory bowel disease is on the rise, with an estimated prevalence of 25,000 cases in Hungary [1]. Ulcerative colitis (UC) is a chronic inflammatory disease of unclear aetiology involving the entire colon. Treatment is primarily medical, but therapy-resistant or complicated cases require surgical intervention aimed at total resection of the colon and rectum, possibly with reconstruction comprising the creation of a pouch in the terminal ileum. Today, proctocolectomy followed by ileal pouch-anal anastomosis (IPAA) is the accepted surgical procedure [2]. Since inflammation that persists for years or decades may involve the risk of malignant transformation without appropriate treatment and gastroenterological follow-up, regular colonoscopies and follow-up for UC patients are crucial. In 2015, the incidence of UC was 21.7/100,000 individuals in Hungary, with an annual mortality rate of 0.01%–0.023%, colorectal cancer being the leading cause of death [3]. However, the possibility of malignant transformation of a chronic inflammation was suspected by German pathologist Rudolf Wirchow as early as the mid-19th century [4].

There have been reports of UC-associated colorectal cancer since the 1920s [5]. The incidence of inflammatory bowel disease-associated colorectal cancer is very low, contributing to only 1%–2% of all colon tumours; however, it is the cause of death in 15% of the UC patient population [6–8]. Unlike sporadic colon cancer, UC-associated colon cancer develops at a younger age and is caused by a malignantly transformed dysplastic lesion associated with chronic colitis [9]. The extent and duration of UC, the involvement of the terminal ileum, the presence of primary sclerosing cholangitis (PSC), and family history may all affect the risk of occurrence [6, 10–13].

Our study objective was to retrospectively assess the occurrence and surgical treatment of UC-associated colorectal cancer cases in our institute and analyse survival data.

## Materials and methods

In the Department of Surgery at the University of Szeged, 39 patients (12 females and 27 males) underwent surgery for UC-associated colorectal cancer between 1 January 2005 and 1 January 2025, with an increasing number of cases annually; they correspond to 25% of all patients who received surgery for UC in our institute in the period specified. The ethical permission number is 175/2023-SZTE RKEB. Their mean age was 55 ± 13.4 years (range: 22–84). Clinical characteristics, risk factors, surgical variables, perioperative outcomes, and long-term survival were assessed retrospectively. Survival was analysed using the Kaplan–Meier method and compared between groups with log-rank tests; curves were displayed with confidence bands.

Because median survival could not be estimated in several strata (the survival curve did not drop below 50% within the follow-up), survival was additionally summarised using the restricted mean survival time (RMST) with a standard error (SE). RMST is the average survival time up to the observed follow-up horizon (i.e., the area under the Kaplan–Meier curve) and provides an intuitive, model-free measure that remains informative under censoring.

To account for multiple comparisons, p-values were adjusted using the Benjamini–Hochberg false discovery rate (FDR) procedure. Given the exploratory nature of the study and limited sample size, both unadjusted and FDR-adjusted p-values were reported and interpreted to balance type I error control with adequate sensitivity to detect potentially relevant signals.

Multivariable Cox regression was not performed because the number of events was low relative to the number of clinically relevant covariates, resulting in an unfavourable events-to-variable ratio that would likely yield unstable, overfitted adjusted estimates.

Statistical analyses were performed with JASP (version 0.95.2). A two-sided significance level of α = 0.05 was used.

## Results

### Preoperative results

Our 39 patients were diagnosed with UC at a mean age of 35.7 ± 16.7 years, and an average of 19.4 ± 12.3 years passed between the diagnosis of UC and the first surgical intervention. It is important to note that 13 of the out-patients did not make regular gastroenterological follow-up visits for decades, a situation associated with significantly worse survival; during our follow-up, 9 of them (69.2%) died. Eight patients (61.5%) underwent surgery at stage IV. Among the 13 patients without a regular follow-up, only 4 required urgent surgery due to obstructive symptoms, meaning that planned surgery was performed in three-quarters of this population. Laparoscopy was conducted in 8 cases, with conversion in 2 patients and open surgery in 3. Two patients underwent IPAA, 7 had resections, and 4 underwent stoma formation.

Regular endoscopies were only performed in 66% of our patients: the lesion was found in the right colon in 8 cases, in the transverse colon in 3 cases, in the left colon in 20 cases, in the rectum in 7 cases, and in the left colon and rectum simultaneously in 1 case. Based on results from the last biopsies before surgery, no malignancy or dysplastic lesion could be confirmed in 10.5% of the cases, and 66% of the patients had active colitis.

Preoperative staging confirmed distant metastases in 12 patients (30.7%) in the form of carcinosis or involvement of the liver, lungs, stomach, or lymph nodes.

PSC was confirmed in 2 cases. Prior biologic therapy had been administered to 6 patients. There was a family history of colorectal cancer in 3 cases. Two of our patients were active smokers. One patient had a history of appendectomy. In our cohort, 21 patients (53.8%) were taking 5-aminosalicylic acid (5-ASA) regularly during the preoperative period.

Three out of the 8 patients with rectal cancer received neoadjuvant oncological treatment in the form of total radiochemotherapy followed by elective surgery.

### Surgical results

#### Emergency surgeries

Indications for emergency procedures (n = 5) included bowel obstruction due to the lesion in the left colon and perforation, and open surgeries were performed, with conversion from laparoscopy in one case (Table 1).

**TABLE 1:** Emergency surgical procedures.

Location	Type of surgery	Method	pTNM	Follow-up/Survival (months)	Stage
Left colic flexure	Subtotal colectomy, partial gastric wall resection	Open surgery	T3N1	179, died	III
Descending colon	Feeding jejunostomy, ileostomy	Open surgery	Carcinosis	2, died	IV
Sigmoid colon	Sigmoidostomy	Conversion	T4M1, peritoneal carcinosis	11, died	IV
Sigmoid colon	Hartmann’s hemicolectomy	Open surgery	T3N2aM1, liver and lung metastases	6, died	IV
Sigmoid colon	Hartmann’s sigmoid resection	Open surgery	T4N2M1, R1, carcinosis	13, died	IV

#### Elective surgeries

Twenty-nine of the elective procedures (n = 34) were performed laparoscopically (Table 2).

**TABLE 2:** Elective surgical procedures: laparoscopic cases (PSC: primary sclerosing cholangitis; HGD: high-grade dysplasia).

Location	Type of surgery	pTNM	Other	Follow-up/Survival (months)	Stage
Caecum	Right hemicolectomy	T1N0	Family history	88	I
Caecum	Right hemicolectomy	T1N0	–	90	I
Right colic flexure	Exploration	Carcinosis	PSC	8, died	IV
Transverse colon	Subtotal colectomy, ileorectostomy	T1N0	–	33	I
Transverse colon	Proctocolectomy, pouch	T4aN1aM1	Recurrence	43	IV
Transverse colon	Proctocolectomy, pouch	T3N0	–	9	II
Left colic flexure	Proctocolectomy, pouch	T2N0	Family history	114, died	I
Left colic flexure	Subtotal colectomy, resection of greater curvature of stomach, ileo-sigmoidostomy	T4N1M1	–	71, died	IV
Sigmoid colon	Proctocolectomy, pouch	T3N0	–	38	II
Rectum	Total colectomy, extirpation	T2N1c	Sigmoid Tis	27, died	III
Rectum	Proctocolectomy, pouch	T1N0	–	157	I
Sigmoid colon	Subtotal colectomy, ileorectostomy	T1N0	–	77	I
Sigmoid colon	Proctocolectomy, pouch	T2, T3N0	Double	5, died	II
Rectum	Proctocolectomy, pouch	T2, T3N1	Triple, R1, ileostomy for pouchitis	75	III
Rectum	Ileostomy	Carcinosis	–	8, died	IV
Rectum	Total colectomy, extirpation	yT0N0, sigmoid: T3N1	Double, NEO	31	III
Sigmoid colon	Proctocolectomy, pouch	T2N0	–	36	I
Sigmoid colon	Hartmann	T4aN1bM1	​	9, died	IV
Left colic flexure	Transverse colostomy	Carcinosis	​	9, died	IV
Left colic flexure	Proctocolectomy, pouch	T4aN0M1	Multiple	14, died	IV
Sigmoid colon	Total colectomy, extirpation	T1N0	Incontinence	15	I
Sigmoid colon	Subtotal colectomy, ileorectostomy	T3N0	​	17	II
Descending colon	Proctocolectomy, pouch	T2N0	Recurrence, extirpation	13	I
Rectum	Extirpation	HGD	–	13	–
Rectum	Proctocolectomy, extirpation	yT3N0	NEO	8	II
Ascending colon	Right hemicolectomy	T4aN2a	–	7	III
Ascending colon	Colectomy, ileorectostomy	T3N2bM1	–	7	IV
Ascending colon	Proctocolectomy, pouch	T1N0	–	6	I
Right colic flexure	Colectomy, ileorectostomy	T3N0	–	6	II

Conversion from laparoscopy was required in 3 cases, and the elective procedure was conventional open surgery in 2 patients (Table 3).

**TABLE 3:** Elective surgical procedures: open and converted cases.

Location	Type of surgery	Method	pTNM	Other	Follow-up/Survival (months)	Stage
Ascending colon	Subtotal colectomy, end ileostomy, mucous fistula, liver resection	Conversion	T4N1M1	PSC	16, died	IV
Sigmoid colon	Total colectomy, end ileostomy	Open surgery	T3N0	–	217	II
Sigmoid colon	Total colectomy, ileorectostomy	Open surgery	T3N0	–	74, died	II
Rectum	Rectal extirpation	Conversion	yT3N0	Smoking, neoadjuvant therapy, ureteric injury	2, died	II
Sigmoid colon	Total colectomy, ileorectostomy	Conversion	T2N0	–	18	I

### Postoperative results and oncological measures

The mean duration of follow-up was 40.2 ± 51.7 months. Among the 11 patients subjected to proctocolectomy and creation of a J-pouch, the diverting ileostomy was closed in 9 patients, it is in progress in 2 cases, and 1 patient died. Subsequently, ileostomy was re-established in 1 case due to severe pouchitis, and the pouch was extirpated in another case because of recurrence in the anastomosis.

As to tumour extent, based on the final histological results, only 7 patients (17.9%) had a T1 lesion and high-grade dysplasia was present in 1 case. Lymph node involvement was confirmed in 17 cases (44.5%), whereas 12 patients (30.7%) showed dissemination with peritoneal carcinosis or involvement of other organs (liver, stomach, urinary bladder, or lungs). Thus, the overall stage distribution of our patient population is as follows: (I) 25.6%, (II) 23%, (III) 15.3%, and (IV) 33.3%. All of the malignancies were adenocarcinomas, including 3 sigillocellular cases and 6 mucinous ones, as well as 1 case each of the anaplastic, adenosquamous, and medullary subtypes.

Histology revealed simultaneous colorectal cancer in several patients, not always known previously – double tumours were described in 5 cases, and triple and multiple tumours in 1 case each. The mean longest diameter of the tumours was 37.8 mm. R1 resection was performed in 2 cases, 1 in the circumferential and 1 in the aboral plane. Microsatellite analysis was conducted in 29 cases, of which 23 were characterised by microsatellite stability. Lynch syndrome was not confirmed. In our material, an average of 39.3 (range: 12–89) lymph nodes were removed during colectomies and proctocolectomies. During our survey, two patients developed the extraintestinal manifestation of PSC, with an advanced stage confirmed in these cases. Adjuvant chemotherapy was administered in 23 cases (58.9%), follow-up was recommended for 13 patients (33.3%), and oncology care was not started in 3 patients because of early death or the patient’s preferences.

We know of 2 cases of recurrence, corresponding to 5.8% of our resected patients.

### Rectal cancer

Neoadjuvant oncological treatment was possible in 3 out of the 8 patients diagnosed with rectal cancer, and the final histological results confirmed complete regression (yT0N0) in one of these cases. Surprisingly, the final histological results revealed double rectal tumours in 1 patient and a simultaneous sigmoid lesion in 3 patients each.

### Survival measures and risk factors

**FIGURE 1:**
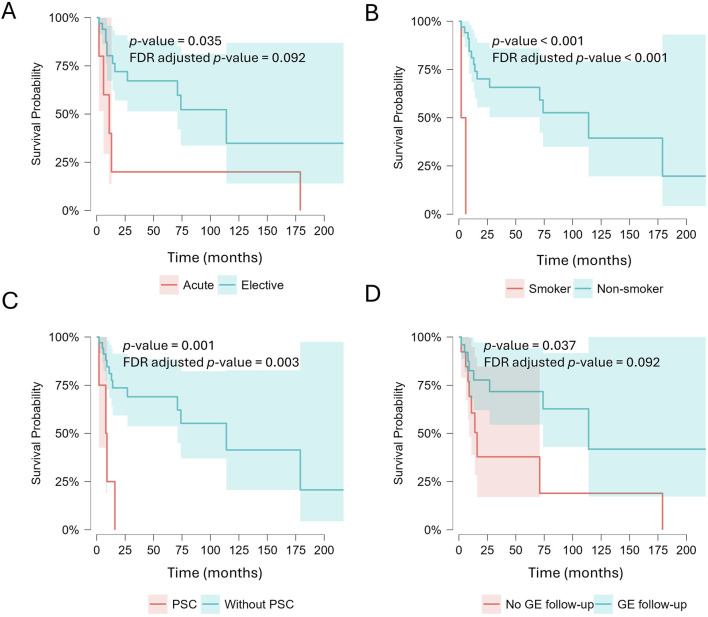
Survival depending on the following factors: **(A)** emergency surgery, **(B)** smoking, **(C)** primary sclerosing cholangitis (PSC), and **(D)** lack of gastroenterological (GE) follow-up.

During the study period, 17 of the 39 patients died. The mean overall survival following surgical treatment was 98.6 months (8.2 years) (Table 4; Figure 1).

**TABLE 4:** Survival measures (RMST: restricted mean survival time; SE: standard error for RMST; p: p-value; FDR: false discovery rate).

Variable	Level	N	Events	RMST	SE	p (log-rank)	FDR- corrected p
Surgical type	Acute	5	5	42.20	30.64	0.035	0.092
	Elective	34	12	110.22	22.35		
Smoking	Smoker	2	2	4.00	1.41	<0.001	<0.001
	Non-smoker	35	14	106.80	18.99		
PSC	With PSC	4	4	8.75	2.48	0.001	0.003
	Without PSC	35	13	111.31	19.36		
Gastroenterological (GE) follow-up	GE follow-up	26	8	124.58	25.23	0.037	0.092
	No GE follow-up	13	9	53.63	22.85		
Duration of UC	Under 10 years	29	14	99.22	19.28	0.963	0.963
	Over 10 years	10	3	134.28	37.53		
Years at baseline	Under 30 years at baseline	19	9	84.09	25.57	0.429	0.669
	Over 30 years at baseline	20	8	116.58	24.90		
Biologic therapy	Received	6	1	181.67	32.26	0.607	0.675
	Not received	33	16	95.60	18.37		
Family history	Positive	6	1	181.67	32.26	0.607	0.675
	Negative	33	16	95.60	18.37		
Colon involvement	Left	21	12	87.95	20.61	0.368	0.604
	Right	11	2	152.69	38.06		
Laparoscopy	Laparoscopy	29	9	109.03	27.05	0.379	0.604
	Open surgery	6	5	81.83	35.21		

Survival was shorter in patients undergoing acute surgery compared with those treated electively (log-rank p = 0.035); however, this difference did not remain statistically significant after FDR correction (FDR-adjusted p = 0.092). Patients undergoing acute surgery also showed a markedly lower RMST (42.2 months) compared with those treated electively (110.2 months), indicating substantially reduced average survival time within the observed follow-up period.

Active smoking was strongly associated with poorer survival. All smokers experienced the event during follow-up, with a markedly reduced RMST (4.0 months) compared with non-smokers (106.8 months). This difference remained statistically significant after FDR correction (log-rank p < 0.001; FDR-adjusted p < 0.001).

Similarly, the presence of PSC was associated with substantially shorter survival (RMST 8.75 vs. 111.3 months), an association that remained significant after FDR correction (log-rank p = 0.001; FDR-adjusted p = 0.003).

Patients with a regular gastroenterological follow-up exhibited longer survival than those without it (RMST 124.6 vs. 53.6 months). Although this difference reached nominal significance in the unadjusted log-rank test (p = 0.037), it did not remain statistically significant after FDR correction (FDR-adjusted p = 0.092).

No statistically significant survival differences were observed for disease duration longer than 10 years, age under 30 years at baseline, prior biologic therapy, family history of colorectal cancer, tumour location within the colon, or surgical technique (laparoscopy vs. open surgery) either before or after FDR correction (all FDR-adjusted p ≥ 0.60). For these variables, RMST estimates and their associated standard errors indicated overlapping survival experiences between groups, consistent with the absence of detectable survival differences within the limits of the available sample size and follow-up duration.

## Discussion

UC-associated colorectal cancer is a highly malignant disease in young patients with a risk of development that gradually increases after 10 years following a UC diagnosis [6, 11, 13]. Carcinogenesis is based on a chronic inflammation-associated progressive dysplastic condition, which differs considerably from the pathophysiology of sporadic colorectal tumours [14].

Between 1 January 2005 and 1 January 2025, a total of 156 patients with UC underwent surgery in our institute, with malignancy confirmed in 25% of them (39 cases) and the number of such cases ever increasing. In view of the risk of malignant transformation of UC, regular colonoscopies and patient follow-up are important. In patients with regular check-ups, Choi et al. detected tumour development at an early stage in 80% of cases [15]. According to the 2017 ECCO (European Crohn’s and Colitis Organisation) guidelines, endoscopy is recommended 8 years after the first symptoms appear [16]. Subsequently, follow-up colonoscopy is recommended annually for high-risk patient populations, whereas low-risk patients require follow-up every 2–3 years; otherwise, follow-up every 5 years is recommended. Based on the ECCO guidelines, among our higher cancer-risk patients, regular surveillance was performed in 1 of 2 PSC cases and in all 3 patients with a positive family history. Among patients with colitis of more than 10 years’ duration, 17 out of 29 participated in regular follow-ups. It is important to note that since thirteen patients did not have regular gastroenterological follow-up visits, regular endoscopies were performed in only 66% of our patients; 66% of the patients had active colitis.

The risk of malignant transformation may be further increased by the occurrence of PSC, which was observed in 2 cases among out-patients in the form of a disseminated, incurable condition and a large liver metastasis [12]. However, a 10-year retrospective Japanese study found no relationship between PSC and cancer risk [17]. Nonetheless, the majority of the literature identifies PSC as a clear risk factor for colorectal cancer in patients with UC [13, 18, 19]. One retrospective study reported a significantly higher risk of colorectal cancer in cases of PSC when associated with IBD. According to the study, the clinical course of this form of PSC also differs from that of isolated PSC [20]. Prior biologic therapy has also been raised as a possible risk factor, but it was not confirmed with the animal experimental model in an Italian study [21]. Six of our patients had received biologic therapy, and the surgical procedure was performed electively with the laparoscopic technique in each case. The extent of UC, the involvement of the terminal ileum, and family history are also possible risk factors [10, 13]. Further, a Dutch survey clearly notes active smoking as a risk factor [22]. Based on data from the literature, prior appendectomy has also been suspected as a risk factor for cancer [23]. In addition, a Chinese summarising study and meta-analysis covering a period of 30 years observed the extent and duration of colitis and geographical factors as independent risk factors. Hungary is among the top countries for incidence of UC-associated colorectal cancer [24]. Anti-inflammatory drugs, especially 5-ASA, play an important role in preventing malignant transformation and in reducing cancer risk [13, 16, 25, 26]. However, the exact mechanism underlying this effect remains unclear, as other anti-inflammatory agents, such as steroids and immunomodulators, do not exhibit a similar effect [27]. 5-ASA is used as first-line therapy for mild to moderate ulcerative colitis. According to the ECCO recommendations, lifelong use may be considered for its chemopreventive effect [28], although its efficacy in combination with other chemopreventive agents is uncertain [16, 28].

During surgical management of UC-associated colorectal cancer, as when surgery is performed for the benign, inflammation “only” type and its complications, removal of the entire target organ, the colon, should be the aim. However, the type and extent of the surgical procedure are determined by the general condition of the patient, the extent of UC, the location of the tumour, the involvement of the sphincter, and the question of oncological treatment. A study from the Far East considers the creation of IPAA and the use of laparoscopy a safe surgical solution for UC-associated colon cancer [29]. Two European studies also referred to IPAA as the gold standard surgical solution [30, 31]. Our study includes 34 elective and 5 emergency surgeries. Among the elective procedures, there were 29 minimally invasive cases and 2 open ones, with conversion being required in 3 cases. Colectomy/proctocolectomy was performed in 21 of our 34 elective cases; of these, IPAA was created in 11 cases. In total, a minimally invasive method was used in 74.3% of the surgeries, and resection was performed in 34 patients (87.1%).

There are 8 cases of rectal cancer among our patients, all in the elective surgery group. Neoadjuvant oncological treatment was possible in 3 cases, and it resulted in 1 confirmed case of complete pathological regression. An Italian working group considers UC-associated rectal tumours a separate entity, emphasising the role of oncological treatment [32].

Lymph node involvement was confirmed in 17 cases (44.5%), whereas 12 patients (30.7%) showed dissemination. During our follow-up, recurrence occurred in 2 cases, that is, in 5.8% of our patients. A Japanese study found young age and stage III to be the main risk factors for recurrence [33].

Results in the literature are divided on UC-associated colorectal cancer survival, but they show a poor prognosis overall [34]. A previous Hungarian epidemiological survey highlighted colorectal cancer among the malignancies of the UC population with unfavourable survival measures, a finding which is consistent with our own experience [3]. The mean survival of 98.6 months observed in our patients was significantly shortened by the following factors: an emergency surgical procedure, smoking, occurrence of PSC, and lack of gastroenterological follow-up.

Several studies found no differences in survival between sporadic and UC-associated cancer groups [34–37]. Other studies, however, attribute a very poor prognosis to UC-associated colorectal cancer [38–40].

The results of our study, although limited by the low number of cases, are consistent with reports that suggest that UC-associated colon tumours represent a group of diseases with a highly aggressive course and early onset. The 25% rate for malignant transformation in our patients is considered very high. It exceeds international results: in two European surveys among patients operated on for UC, colorectal cancer occurred in 12% and 16.9% of cases, respectively [31, 41]. Several of our patients had a disseminated disease at the time of surgery, but the percentage of curative surgeries was also high, thus impacting long-term survival considerably. Our observation is supported by a Hungarian survey that notes unfavourable results for survival [3], which, based on our study, is significantly shortened by PSC, lack of follow-up, smoking, and emergency surgery. Based on our experience, it is especially important for UC patients to receive close gastroenterological follow-ups in specialised centres and undergo regular colonoscopies and for staff to evaluate biopsy samples properly and perform appropriate surgical procedures in due time, preferably proctocolectomy and creation of IPAA with a minimally invasive method. Because of the incidental finding of malignancy and multilocular lesions, central ligations are recommended during routine resections for UC.

European results for survival outcomes in UC-associated malignancies are heterogeneous, with most studies reporting unfavourable outcomes, thus confirmed by our present observations. However, our study is limited by the low number of cases, as well as by the lack of survival data for the tumour-free UC population and for patients with sporadic colorectal carcinoma [31, 41].

### Limitations

Importantly, given the limited number of events (17 deaths among 39 patients) and the small size of certain subgroups, both unadjusted and FDR-adjusted p-values should be interpreted cautiously even when they fall well below conventional significance thresholds because sparse-event strata can inflate apparent effect sizes and reduce the stability and generalisability of estimates. Therefore, these findings should be considered hypothesis-generating rather than definitive.

## Data Availability

The raw data supporting the conclusions of this article will be made available by the authors, without undue reservation.
